# DNA barcoding cannot discriminate between *Sardinella tawilis* and *S. hualiensis* (Clupeiformes: Clupeidae)

**DOI:** 10.1080/23802359.2019.1638839

**Published:** 2019-07-13

**Authors:** Alison Faye O. Chan, Adrian U. Luczon, Ian Kendrich C. Fontanilla, Perry S. Ong, Mudjekeewis D. Santos, Demian A. Willette, Jonas P. Quilang

**Affiliations:** a Molecular Population Genetics Laboratory, Institute of Biology, College of Science, University of the Philippines, Diliman, Quezon City, Philippines;; b DNA Barcoding Laboratory, Institute of Biology, College of Science, University of the Philippines, Diliman, Quezon City, Philippines;; c National Fisheries Research and Development Institute, Quezon City, Philippines;; d Biology Department, Loyola Marymount University, Los Angeles, CA, USA

**Keywords:** COI, DNA barcoding, RAG1, *Sardinella hualiensis*, *Sardinella tawilis*

## Abstract

*Sardinella tawilis*, the only known freshwater sardinella in the world, is endemic to Taal Lake, Philippines. Previous studies found the Taiwan sardinella, *S. hualiensis*, to be morphologically very similar to *S. tawilis* and identified it as the marine sister species of *S. tawilis*. In this study, DNA barcoding using the mitochondrial cytochrome *c* oxidase I (COI) gene was carried out to analyze species demarcation in the *Sardinella* genus, focusing primarily on the relationship between *S. tawilis* and *S. hualiensis*. The neighbour-joining (NJ) tree that was constructed using Kimura 2-parameter (K2P) model showed a single clade for the two species with 100% bootstrap support. K2P interspecific genetic divergence ranged from 0% to 0.522%, which is clearly below the suggested 3–3.5% cutoff for species discrimination. Recombination activating gene 1 (RAG1), mitochondrial control region (CR), cytochrome b, 16S rRNA, and S7 markers were used to further validate the results. *Sardinella tawilis* and *S. hualiensis* clustered together with a bootstrap support of 99–100% in each of the NJ trees. Low interspecific genetic distances between *S. tawilis* and *S. hualiensis* for all the markers except CR could be attributed to incipient allopatric speciation.

## Introduction

*Sardinella tawilis* (Herre 1927), a commercially important fish in the Philippines, is the only known freshwater sardinella in the world. It is endemic to Taal Lake, a crater lake produced as a result of a series of powerful volcanic eruptions and other geological activities that eventually led to basin formation (Hargrove 1991; Ramos 2002). Taal Lake once had a larger and wider channel that drained into Balayan Bay of the South China Sea (Hargrove 1991). The volcanic eruptions in 1754 closed off what is now the Taal Lake and the saltwater connection was shut off with the *Sardinella* species and other fishes of marine origin inhabiting the lake trapped inside. The lake is currently connected to the West Philippine Sea by the very narrow Pansipit River. Prior to the eruption in 1754, Taal Lake was brackish and since then it gradually became fresh (Herre 1927; Hargrove 1991). There is an interest in the origin and evolutionary biology of *S. tawilis* as well as in its conservation since overfishing has become a problem and the stock is continuously being depleted. The species was listed as endangered by the International Union for Conservation of Nature (Santos et al. 2018).

Samonte et al. (2000) identified *Sardinella albella* as the closest marine relative of *S. tawilis* based on mitochondrial control region (CR) interspecific distances of 1.3–3.5%. They also suggested that *S. tawilis* and *S. albella* could be ecomorphs of the same species since the maximum parsimony and neighbour-joining (NJ) trees they constructed grouped the two species together. Samonte et al. (2009) further supported this through multivariate analyses of meristic and morphometric characters. However, Quilang et al. (2011), using mitochondrial cytochrome *c* oxidase subunit I (COI) gene, showed that *S. tawilis* is distinctly separate from its other marine relatives with genetic divergence ranging from 16.4% to 19.3%. It was suggested that a misidentification might have taken place since the single specimen of *S. albella* acquired by Samonte et al. (2000) from a fish vendor in Lemery, Batangas may possibly be *S. tawilis* since the location is very close to Taal Lake.

The collection of *Sardinella hualiensis* (Chu and Tsai 1958) from Cagayan province in northern Philippines by Willette et al. (2011) was the first report of the Taiwan sardinella in the country. Based on the NJ tree of the mitochondrial 16S rRNA gene constructed using the Kimura 2-parameter (K2P) model, a single clade containing *S. hualiensis* from both Taiwan and the Philippines was formed, which was supported by a bootstrap value of 91% (Willette et al. 2011). The morphometric and meristic data were within the ranges described by Whitehead (1985), except for the gill raker count, which was attributed to plasticity due to their natal environment (Willette et al. 2011). Willette et al. (2014) identified *S. hualiensis* as the marine sister species of *S. tawilis* using 16S rRNA (genetic distance = 0.1%), cytb (0.1%), and S7 (0.3%) markers.

Owing to the similar and overlapping morphology and meristics of *S. hualiensis* with *S. tawilis* (Willette et al. 2011), further molecular analysis was required to determine the relationship between these two species. Better understanding of the genetic status of *S. tawilis* could help in sustainability and management studies for this fish species, which is the main source of income and livelihood for many of the locals in the Taal Lake area. This study aimed to analyze species demarcation in the *Sardinella* genus using the mitochondrial COI gene, focusing primarily on *S. tawilis* and *S. hualiensis.* Nuclear recombination activating gene 1 (RAG1), cytochrome b (cytb), 16S rRNA, CR, and nDNA S7 were then used to further validate the findings.

## Materials and methods

Muscle tissues were obtained from 12 fresh specimens of *S. tawilis* from Taal Lake at Talisay Fish Port, Batangas, Philippines (14.09°N, 121.02°E) and 10 individuals of *S. hualiensis*, which were provided by the National Fisheries Research and Development Institute (NFRDI). Five of the 10 *S. hualiensis* came from Santa Ana, Cagayan, Philippines (8°30′N, 122°8′E) and the other five from Yilan County, Taiwan (24°34′N, 121°52′E). *Sardinella tawilis* and *S. hualiensis* specimens were kept as vouchers at the Institute of Biology, University of the Philippines Diliman and NFRDI, respectively.

DNA was extracted from the muscle tissues using Promega Wizard^®^ Genomic DNA Purification Kit (Madison, WI). Polymerase chain reaction (PCR) was performed in 15 μl volumes containing 0.3 μl of dNTP (0.05 mM), 0.75 μl of each primer (0.1 mM), 1.5 μl of 10× PCR buffer, 3 μl of 5× Q buffer, 0.3 μl of MgCl (25 mM) buffer, 0.075 μl of *Taq* polymerase, 7.125 μl of ultrapure water, and 1.2–2.4 μl of DNA template.

The following gene regions were PCR amplified with corresponding primers:

1. COI (636 nucleotides) – FishF2 and FishR1 (Ward et al. 2005)
2. RAG1 (1504 nucleotides) – nested PCR: first round RAG1-2510F and RAG1-4063R (Li and Orti 2007); second round RAG1-2533F (Lopez et al. 2004) and RAG1-4063R (Li and Orti 2007); first fragment third round RAG1-2533F and RAG1-3261R (Li and Orti 2007); second fragment third round RAG1-3098F (Li and Orti 2007) and RAG1-4063R
3. CR (433 bp) – LCHACR04 (5′-AACTCCCAAAGCTAGGATTC-3′) and CHACR01H (5′-GGCCCATCTTAACATCTTCA-3′) designed in this study
4. cytb (405 bp) – LCHACB02 and CHACB02H (Santos et al. 2019)
5. 16S (520 bp) – 16Sar and 16Sbr (Palumbi 1996)
6. S7 (708 bp) – nested PCR: first round S7RPEX1F and S7PEX2R (Chow and Hazama 1998); second round 1F.2 and 2R.67 (Chow and Hazama 1998)

The purified PCR products were sent to 1st Base in Selangor Darul Ehsan, Malaysia for bidirectional sequencing. Forward and reverse sequences were edited using Staden Package (Staden et al. 2000) and Bioedit (Hall 1999) to produce a final consensus sequence. Sequences of other *Sardinella* species were downloaded from GenBank and used in the analysis. MEGA 6 (Tamura et al. 2013) was used to compute the average pairwise comparisons of sequence differences between and within species. NJ trees using K2P model were constructed with bootstrap analysis of 1000 replicates (Kimura 1980; Saitou and Nei 1987). PAUP* (Swofford 2003) was used to identify haplotypes.

COI sequences and other metadata were submitted to Barcode of Life Data Systems. All sequences were submitted to GenBank and were assigned accession numbers MK585631-MK585652, MK575290-MK575358, and MK675086-MK675088.

## Results

A total of 94 sequences were generated in this study, which included 22 barcodes using the mitochondrial COI gene, 24 using nuclear RAG1, 12 using 16S rRNA, 12 using cytb, 12 using CR, and 12 using S7. The 10 *S. hualiensis* barcodes were the first reported for this species.

**Figure 1.**
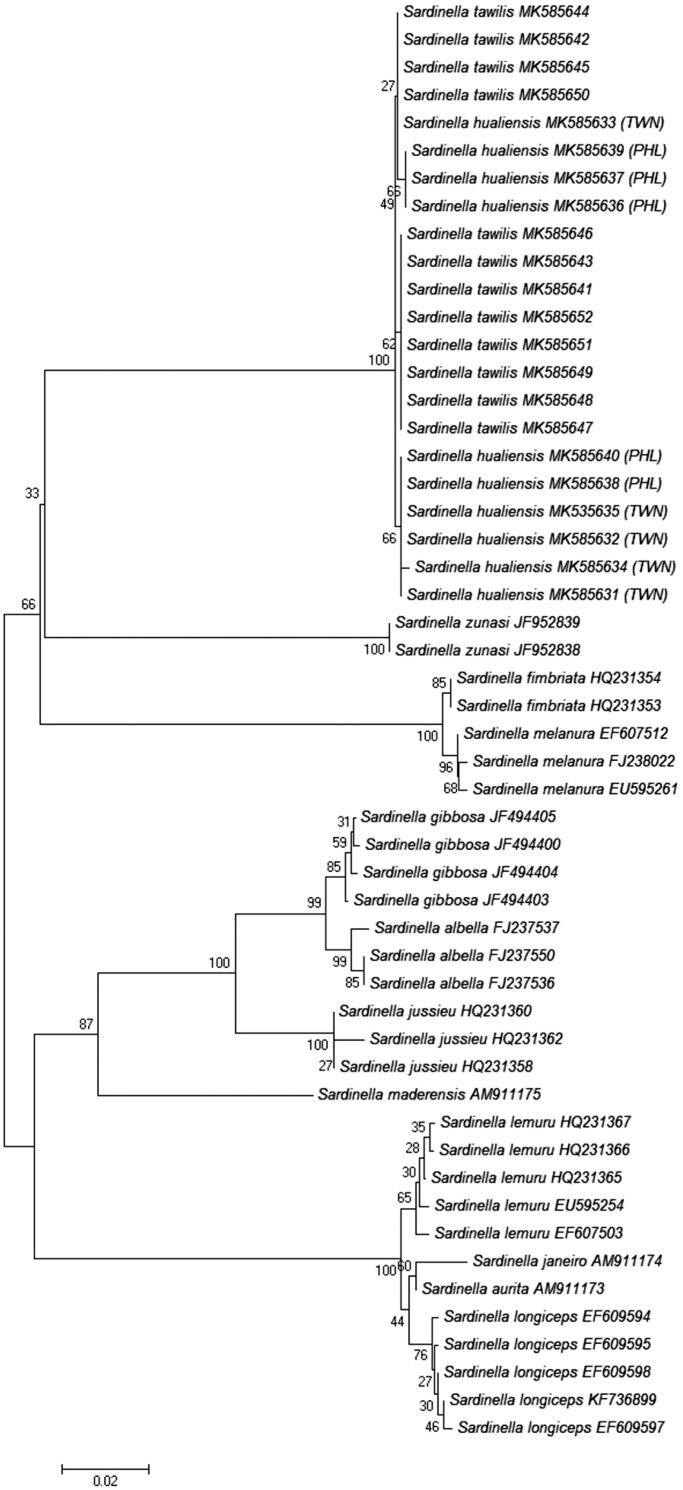
Unrooted neighbour-joining tree using Kimura 2-Parameter (K2P) genetic distances of 52 COI sequences from 13 *Sardinella* species. Bootstrap values based on 1000 replicates are shown at nodes. The *S. tawilis* and *S. hualiensis* sequences were generated from this study. All other sequences were downloaded from GenBank with their designated accession numbers. Scale bar represents two nucleotide changes per 100 nucleotides. PHL: Philippines; TWN: Taiwan.

**Figure 2.**
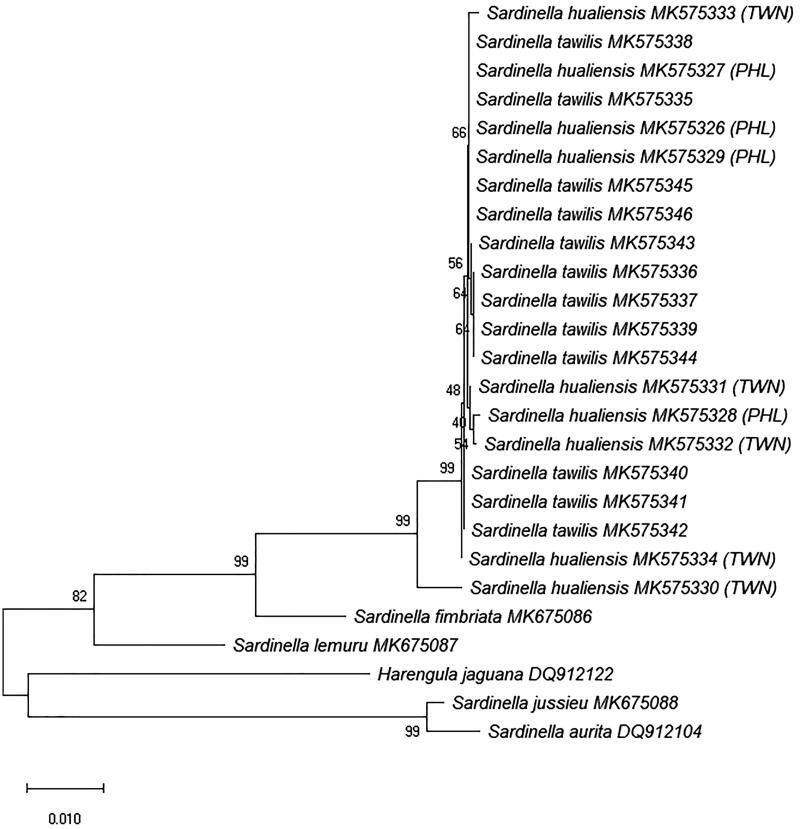
Unrooted neighbour-joining tree using Kimura 2-Parameter (K2P) genetic distances of 26 RAG1 sequences from six *Sardinella* species and outgroup *Harengula jaguana*. Bootstrap values based on 1000 replicates are shown at nodes. The *S. tawilis* and *S. hualiensis* sequences were generated from this study while the outgroup was downloaded from GenBank with designated accession numbers. Scale bar represents one nucleotide change per 100 nucleotides. PHL: Philippines; TWN: Taiwan.

The interspecific distances between *S. tawilis* and *S. hualiensis* were very low for all genetic markers except for CR (3.4429–6.0485%, mean = 6.0485%), which is the most variable region in the mitochondrial DNA and is used more often to discriminate populations and not between species. The minimum K2P genetic divergence of all other gene regions aside from CR was zero: COI (0–0.559%, mean = 0.286%), RAG1 (0–1.171%, mean = 0.191%), 16S (0–0.587%, mean = 0.133%), cytb (0–0.521%, mean = 0.118%), S7 (0–0.474%, mean = 0.191%). Based on COI (Figure 1), RAG1 (Figure 2), 16S, CR, cytb, and S7 NJ trees (figures not shown) generated, *S. tawilis* and *S. hualiensis* clustered together with a bootstrap support of 99–100%, clearly showing that these two species are the most closely related.

There were also shared haplotypes among *S. hualiensis* from the Philippines, *S. hualiensis* from Taiwan, and *S. tawilis* in all gene regions other than mitochondrial control region (Table 1).

**Table 1.** Summary of sample sizes (*n*), number of haplotypes, and number of private haplotypes for COI, RAG1, 16S, Control Region, Cyt *b*, and S7 for *Sardinella hualiensis* from Taiwan (TWN) and Philippines (PHL) and *S. tawilis.*

Gene/species	*N*	GenBank accession nos.	No. of haplotypes	No. of private haplotypes
COI				
*S. hualiensis* TWN	5	MK585631-35	3	1
*S. hualiensis* PHL	5	MK585636-40	3	2
*S. tawilis*	17	MK585641-52, HQ231368-72	2	1
RAG1				
*S. hualiensis* TWN	5	MK575330-34	5	5
*S. hualiensis* PHL	4	MK575326-29	2	1
*S. tawilis*	12	MK575335-46	5	4
16S				
*S. hualiensis* TWN	10	JN580482-91	2	1
*S. hualiensis* PHL	6	JN580476-81	2	1
*S. tawilis*	18	MK575290-301, KC951487-92	5	4
Control Region				
*S. hualiensis* TWN	6	KC951511-16	6	6
*S. hualiensis* PHL	6	KC951517-22	5	5
*S. tawilis*	18	MK575302-13, KC951505-10	14	14
Cyt *b*				
*S. hualiensis* TWN	4	KC951529-32	2	1
*S. hualiensis* PHL	6	KC951523-28	2	1
*S. tawilis*	18	MK575314-25, KC951533-38	3	2
S7				
*S. hualiensis* TWN	6	KC951475-80	4	3
*S. hualiensis* PHL	3	KC951471-74	3	1
*S. tawilis*	18	MK575347-58, KC951481-86	5	3

The sequences included in this analysis were those generated in this study and those generated by Quilang et al. (2011), Willette et al. (2011), and Willette et al. (2014).

## Discussion

In this study, mitochondrial COI and nuclear RAG1 markers were unable to distinguish between *S. tawilis* and *S. hualiensis*. The interspecific K2P genetic distances based on the COI sequences ranged from 0% to 0.559% (mean = 0.286%), which fall below the 3–3.5% threshold to differentiate species (Hebert et al. 2003; Ward et al. 2005; Lara et al. 2010). The low mean genetic divergence of <1% (0.191%) from RAG1 sequences between *S. tawilis* and *S. hualiensis* provide additional evidence in supporting the COI results, which suggests that *S. tawilis* and *S. hualiensis* belong only to a single species. Nuclear barcodes supplement the mitochondrial barcodes and allow greater reliability to the identification and groupings of species (Dasmahapatra and Mallet 2006). Using nuclear markers in addition to mitochondrial gene markers could determine introgressive hybridization between species and further resolve findings (Lara et al. 2010).

*Sardinella tawilis* and *S. hualiensis* clustered together in the NJ trees, with a bootstrap support of 99–100%, presenting these two species as the most closely related. However, what is interesting is that *S. tawilis* is a freshwater species, while *S. hualiensis* is a marine species, and these two can be found in areas quite distant from each other. *Sardinella tawilis* is geographically isolated within Taal Lake.

A particularly noteworthy finding is the 0 genetic distance between *S. tawilis* and one Taiwan specimen of *S. hualiensis* (MK585633) based on COI data. It is possible that *S. tawilis* and *S. hualiensis* most likely shared a recent common ancestor unless they are conspecific. As Willette et al. (2011) has pointed out, it has not yet been determined whether *S. hualiensis* originated from the Philippines or from Taiwan. There are factors that limit and complicate the dispersal between Taiwan and the Philippines, such as the unfavourable environment of the Luzon Strait that separates the two locations and the interacting currents that limit the dispersal of larvae. It was hypothesized that the Taiwan, Batanes, and Babuyan Islands were possibly used as stepping stones or stopovers as the *S. hualiensis* crossed the Luzon Strait (Willette et al. 2011). But, to date, the presence of *S. hualiensis* has never been reported in Balayan Bay or in West Philippine Sea. What is clear is that *S. albella* is definitely not the closest marine relative to *S. tawilis*, contrasting with what Samonte et al. (2000) and Samonte et al. (2009) claimed in their work. When genetic divergence was compared between *S. hualiensis* and other species of *Sardinella*, *S. tawilis* and *S. hualiensis* are the most closely related. For all others, the genetic distances ranged from 16.063% to 20.250%.

Although DNA barcoding would consider *S. tawilis* and *S. hualiensis* as belonging to one species on the basis of low interspecific genetic distances, Willette et al. (2014) consider *S. tawilis* and *S. hualiensis* as separate sister species from the perspective of the biological and evolutionary species concepts due to their geographic and reproductive isolation hindering natural interbreeding, the phylogenetic species concept due to some diagnosable morphological differences despite being more similar to each other than other *Sardinella* species, and the physiologic evolution of the osmoregulatory mechanism from freshwater to marine. It is possible that they are still in the process of incipient allopatric speciation, which would explain the low interspecific genetic distance between *S. tawilis* and *S. hualiensis* despite geographic separation. Geographic isolations are capable of creating species over evolutionary time scales. Incipient allopatric vicariant speciation could possibly explain the low interspecific genetic distance between *S. tawilis* and *S. hualiensis.* They could be one species that started to differentiate after isolation occurred in 1754 when the Taal Lake’s saltwater connection was shut off (Hargrove 1991).
